# Comparison of glycemic improvement between intermittent calorie restriction and continuous calorie restriction in diabetic mice

**DOI:** 10.1186/s12986-019-0388-x

**Published:** 2019-08-28

**Authors:** Siying Wei, Jingyu Zhao, Meijuan Bai, Chenchen Li, Lingling Zhang, Yan Chen

**Affiliations:** 1 CAS Key Laboratory of Nutrition, Metabolism and Food Safety, Shanghai Institute of Nutrition and Health, Shanghai Institutes for Biological Sciences, University of Chinese Academy of Sciences, Chinese Academy of Sciences, 320 Yueyang Rd, Shanghai, 200031 China; 2 School of Life Sciences and Technology, Shanghai Tech University, Shanghai, 200031 China

**Keywords:** Diabetes, Intermittent fasting, Fasting-mimicking diet, Calorie restriction, Insulin sensitivity

## Abstract

**Background:**

Calorie restriction (CR) has been well proved to be a powerful tool to improve metabolic health associated with aging; and many types of CR have been proposed. Intermittent CR has become a trend in recent years due to its better compliance than continuous CR every day. However, there are few studies that directly compare the interventional activity of intermittent CR vs continuous CR in metabolic disorders such as diabetes.

**Methods:**

In this study, we analyzed two protocols of intermittent CR with the calorie-matched continuous CR in two diabetic mouse models including *db/db* and streptozotocin-treated mice. Intermittent CR was carried out by a fasting-mimicking diet (FMD, with 30% calorie intake of the control per day) for 2 days or 5 days (i.e., 2–5 or 5–9 regimes followed by free eating for 5 or 9 days respectively).

**Results:**

In the two diabetic mouse models, both intermittent CR and continuous CR significantly reduced fasting blood glucose level and improved insulin sensitivity. However, intermittent CR performed significantly better than continuous CR in improving glycemic control and insulin sensitivity in *db/db* mice. In addition, intermittent CR improved the glucose homeostasis of the *db/db* mice without causing loss of body weight. Analyses with the pancreatic islets reveal that intermittent CR profoundly elevated the number of insulin-positive cells in both diabetic mouse models.

**Conclusions:**

Our study indicated that both intermittent CR and continuous CR can lower fasting blood glucose level in the diabetic mice, while intermittent CR is better than the latter in improving glucose homeostasis in *db/db* mice.

**Electronic supplementary material:**

The online version of this article (10.1186/s12986-019-0388-x) contains supplementary material, which is available to authorized users.

## Introduction

Weight loss, long considered as a key strategy to ameliorate the progression of type 2 diabetes and calorie restriction (CR), has been widely used to reduce obesity [1, 2]. There are numerous types and regimes of CR used in both animal and human studies. Intermittent CR or intermittent fasting (IF) has gained a lot of popularity in recent years as it is easily acceptable to patients [3]. Intermittent CR refers to a dietary pattern of extended time period of fasting with low or no energy intake (e.g., 16–48 h), followed by a period of normal food intake, on a recurring basis [4]. It is believed that intermittent CR can induce a metabolic switch from lipid/cholesterol synthesis and fat storage to mobilization of fat through fatty acid oxidation and fatty acid-derived ketone bodies which have the potential to improve body composition in overweight individuals [5]. In laboratory animals, intermittent CR has profound beneficial effects on metabolic health, slow down disease processes, and improve age-related disorders including diabetes, cancers, cardiovascular disease, and neurodegenerative disorders [4]. The common concept in the field is that CR-mediated loss of obesity is the primary cause of glycemic improvement in diabetes. However, it was found that calorie restriction is a significant factor in glycemic control in obese type 2 diabetic patients, in addition to the magnitude of weight loss [6]. In this study, we investigate whether CR-mediated weight loss is associated with glycemic control in diabetic mice.

To increase the compliance of intermittent CR in humans, Dr. Longo’s group has proposed the concept of fasting-mimicking diet (FMD) instead of simple fasting [7]. Application of FMD for 4 days every 2 months in middle-aged mice led to a reduction in cancer incidence, extension in lifespan, and decrease in visceral fat [7]. Additionally, intermittent use of FMD improved demyelination in a murine model of experimental autoimmune encephalomyelitis [8]. The combination of FMD with chemotherapy delayed the progression of melanoma and breast cancers via boosting T cell-mediated cytotoxicity [9]. Recently, a human experiment with 100 healthy subjects indicated that administration of a FMD low in calories, sugar, and protein, but high in unsaturated fat, had favorable effects on body mass index and blood pressure, in addition to improvements in blood levels of fasting glucose, C-reactive protein, insulin-like growth factors-1 (IGF-1), triglycerides, total cholesterol, and low-density lipoprotein cholesterol in subjects at risk for diseases [10]. Lately, FMD was shown to improve glucose homeostasis, promote β cell generation, and restore insulin secretion in type 1 and type 2 diabetic mouse models [11], heralding the potential use of the FMD to reverse the pathogenesis of diabetes.

Although a plethora of studies have investigated the interventional activities of different modes of CR, there are few reports that directly compare intermittent CR vs continuous CR in the same study. In this study, we compared the interventional efficacy of intermittent CR vs continuous CR in both type 1 and type 2 diabetic mouse models. Intriguingly, we found that while both intermittent CR and continuous CR could improve glycemic control in the mice, intermittent CR performed better than continuous CR in certain aspects.

## Experimental methods

### Mouse model

Four to six-week-old male C57BL/ksJ-db (*db/db*) mice and normal C57BL/6 mice purchased from SLAC (Shanghai, China) were maintained in pathogen-free conditions and kept on a 12 h light/dark cycle at the Institute for Nutritional Sciences. All mice were fed until they were 10-weeks-old and then weighed at the beginning of the study and randomly allocated to experimental groups. The control group was fed with normal chow with free access to food and water. Two regimes of intermittent CR (2–5 and 5–9) was used with calorie restriction for 2 or 5 days followed by free access to normal chow for 5 days or 9 days respectively. Intermittent CR was administered by a FMD that contained low level of protein and high level of fiber [12]. During the use of FMD, the calorie intake was 30% of the daily calorie intake of the normal control group. We also included two groups of continuous CR that matched the total calculated calorie intake of the 2–5 and 5–9 intermittent FMD groups (with ~ 81% and ~ 76% of daily calorie intake of the normal control group respectively). CR group was fed with normal chow as control group. Before the beginning of the experiment, we recorded the average food intake per day per mice for one week. Based on the recorded value, we calculated the amount of FMD (1/3 cal intake per day during the fasting period) and that of continuous CR. In addition, the mice of intermittent FMD and continuous CR groups were caged individually to make sure the food intake was well controlled. The food intake of the mice was carefully recorded to make sure that the calorie intake of intermittent FMD group and continuous calorie restriction (CR) group were strictly controlled throughout the entire period of the experiment (Additional file 1: Figure S1). The animal experiments were conducted in accordance with the guidelines of the Institutional Animal Care and Use Committee of the Institute for Nutritional Sciences, Shanghai Institutes for Biological Sciences (SIBS), Chinese Academy of Sciences (CAS) with an approval number 2010-AN-8. For inducing T1D, male C57BL/6 mice were fasted 4 h prior to STZ injection (40 mg/kg). STZ was purchased from Sigma-Aldrich (Cat. No. S0130) and dissolved in sodium citrate buffer (pH 4.5, freshly prepared) at a concentration of 4 mg/ml immediately prior to use and STZ solution was administered intraperitoneally within 5 min according to mice weight. Mice were then returned to their cage and 10% sucrose water was provided to prevent hypoglycemia after STZ injection. Two weeks after STZ injection, all mice became hyperglycemia (> 10 mmol/ml). For T2D, four to six-week-old male C57BL/ksJ-db (*db/db*) mice were purchased from SLAC (Shanghai, China). All mice were fed to become hyperglycemia (> 10 mmol/ml) after 2 or 3 weeks before intervention.

### Fasting mimicking diet

The FMD used in this study (Gembynear Nutrition Bar or Zhenbainian in Chinese) was kindly provided by Beijing Winlife Research Institute of Nutrition, Health, Food Science, and Technology (Beijing, China). The nutritional composition of the FMD was provided as previously reported [12]. During the use of FMD, all mice were supplied with fresh food in the morning (~ 9:00 a.m.). The mice usually consumed the supplied food within the first few hours.

### Blood glucose and insulin measurement

All mice were fasted for 6 h in the morning before blood glucose measurements. Blood glucose was measured through the tail vein by the use of the OneTouch UltraEasy Blood Glucose Monitoring System (Lifescan, Milpitas, CA, USA). Serum insulin levels were determined by a murine enzyme-linked immunosorbent assay kit (Shanghai Enzyme-linked Biotechnology Co., Shanghai, China). Whole blood was withdrawn from the orbital sinus, and plasma was separated by centrifugation at 3400 rpm for 15 min in EDTA-K2-treated microtubes (Kangjian Medical, China).

### Glucose tolerance testing (GTT) and insulin tolerance testing (ITT)

Mice were caged individually and fasted for 4 h for ITT in the morning and fasted overnight for GTT. Glucose (1 g/kg) or insulin (3 unit/kg) was injected intraperitoneally. Blood glucose levels were measured from 0 min to 120 min after injection.

### Immunofluorescence analysis

Mice pancreas samples were collected then rinsed in PBS and fixed in 4% paraformaldehyde overnight before being dehydrated and embedded into paraffin. After that, samples were sectioned into 4 μm slices. The immunofluorescence staining was performed as previously described [12]. The following primary antibodies were used: anti-insulin (cat. no. C27C9, Cell Signaling Technology, Danvers, MA, USA), anti-glucagon (cat. no. ab10988, Abcam, Cambridge, UK). The nucleus was stained with Hoechst 33342 (Eugene Oregon, USA). The images were captured by a 40x objective with an LSM 510 confocal microscope (Zeiss, Jena, Germany).

### Measurement of serum and hepatic parameters

Mice were euthanized, and the blood of the mice was immediately collected from mice orbital sinus into EDTA-K2-treated microtubes (Kangjian Medical, China). After that, the microtubes were centrifuged at 3400 rpm for 15 min, and the blood serum was then collected. Except for those for immediate use, all serum samples were stored at − 80 °C. Serum levels of aspartate transaminase (AST) and alanine transaminase (ALT) were determined by an AST/ALT determination kit (ShenSuo UNF, China). Serum levels of triglycerides (TG) and total cholesterol (TC) were determined by colorimetric methods with the corresponding kits (ShenSuo UNF, China). All of these assays were performed as previously described [12].

### Antibodies and immunoblotting

The antibodies against total AKT and phosphorylated AKT were from Cell Signaling Technology (Danvers, MA, USA). The protocols for immunoblotting have been described previously [13].

### Statistical analysis

All data were expressed as the mean ± SEM. Significant differences were assessed by one-way ANOVA followed by the Student-Newman-Keuls test where appropriate or by two-tailed *Student’*s t test. Statistical tests were performed using Microsoft Excel (Microsoft, Redmond, WA, USA), R v3.3.2, or Prism6 (GraphPad Software, La Jolla, CA, USA) where appropriate.

## Results

### Comparison of intermittent CR and continuous CR in *db/db* mice

**Fig. 1:**
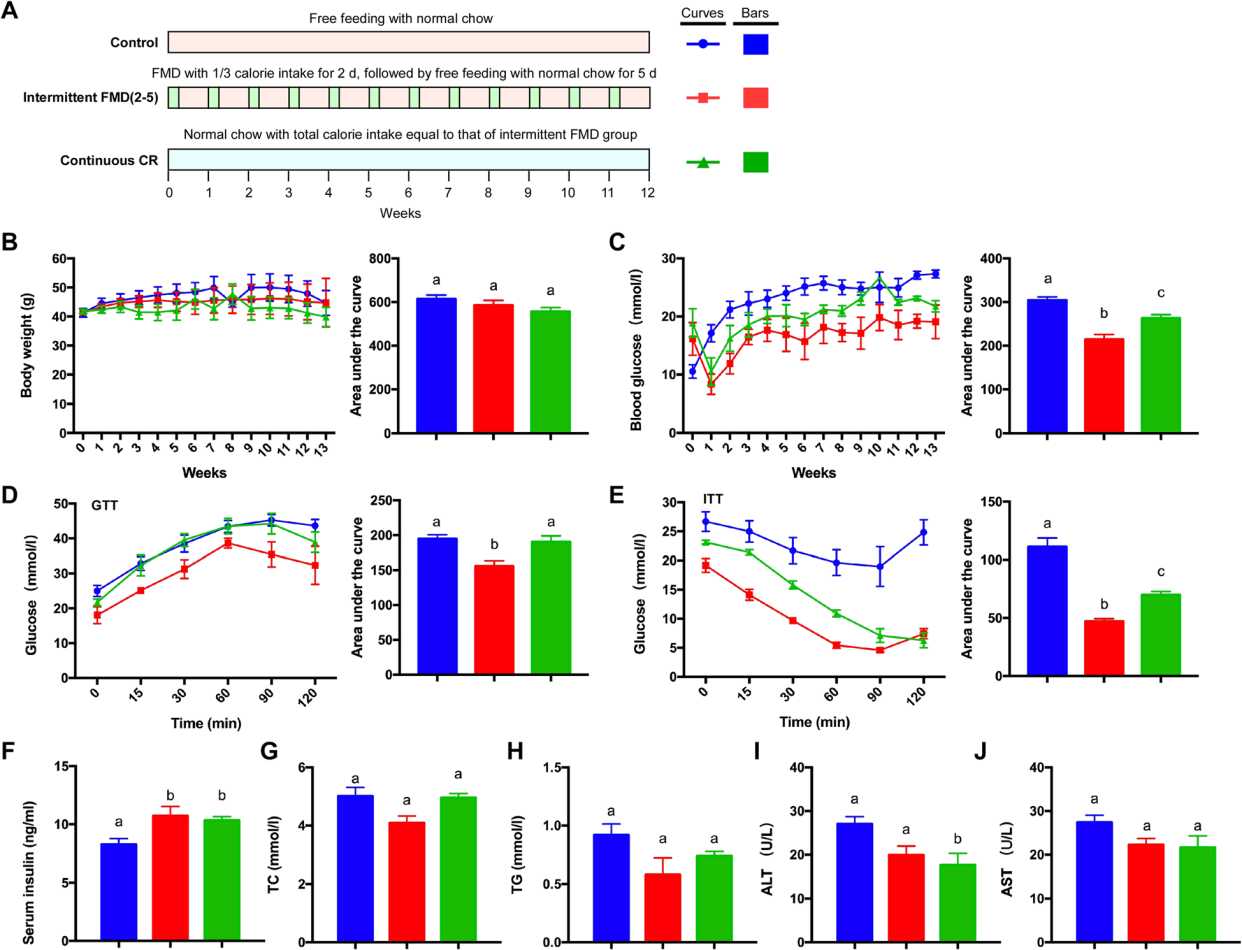
Comparison of 2–5 regime of intermittent fasting vs matched continuous CR in *db/db* mice. (**a**) Experimental design scheme with *db/db* mice (*n* = 5 for each group). (**b**) Body weight. The area under the curve is shown on the right. (**c**) Blood glucose levels. Blood samples were collected on the last day of each cycle. Mice were fasted for 6 h (morning fasting) before glucose measurements. The area under the curve is shown on the right. (**d**, **e**) Glucose tolerance test (GTT) and insulin tolerance test (ITT) at week 13. The area under the curve is shown on the right. (**f**) Serum insulin levels under fed state. (**g**, **h**) Levels of total triglycerides and total cholesterol in the blood. (**i**, **j**) Blood ALT and AST levels of the mice. Data are expressed as the mean ± SEM, n = 5 per group. Significant differences were assessed by one-way ANOVA followed by the Student-Newman-Keuls test where appropriate

We first compared the interventional effect of 2–5 regime of intermittent CR vs continuous CR in *db/db* mice that had severe type 2 diabetes due to extreme obesity caused by deficiency of leptin receptors. For implementation of intermittent CR, we used a fasting-mimicking diet (FMD) that is vegetable-based, and lower in protein and carbohydrates compared to normal chow [12]. The *db/db* mice were divided into three groups (Fig. 1a). The control group had ad libitum (AL) access to normal chow. The intermittent FMD group was exposed to FMD for 2 days (with 1/3 cal intake of the control) and ad libitum diet for 5 days per week. The matched continuous CR group had normal chow with total energy intake equal to the calculated intermittent FMD group (~ 81% energy intake of the control group per day). During the 13 weeks of intervention, the body weight of the three groups of mice was not significantly different from each other (Fig. 1b). The elevated fasting blood glucose level in *db/db* mice was significantly reduced by both intermittent FMD and continuous CR (Fig. 1c). Analysis with the area under the curve revealed that the cumulative fasting blood glucose level in the intermittent FMD group was lower than that of continuous CR group (Fig. 1c). Both GTT and ITT were performed at the end of the experiment with the animals. The glucose tolerance of the mice was significantly improved by intermittent FMD, but not by continuous CR (Fig. 1d). Insulin sensitivity was improved by both intermittent FMD and continuous CR, while intermittent FMD performed better than continuous CR (Fig. 1E). In addition, the blood insulin level was significantly elevated by both intermittent FMD and continuous CR (Fig. 1f). However, the serum levels of total cholesterol (TC) and triglyceride (TG) were not altered by either type of CR (Fig. 1g and h). Only continuous CR reduced the serum level of ALT (Fig. 1i). However, the serum level of AST was not altered by either type of fasting (Fig. 1j).

**Fig. 2:**
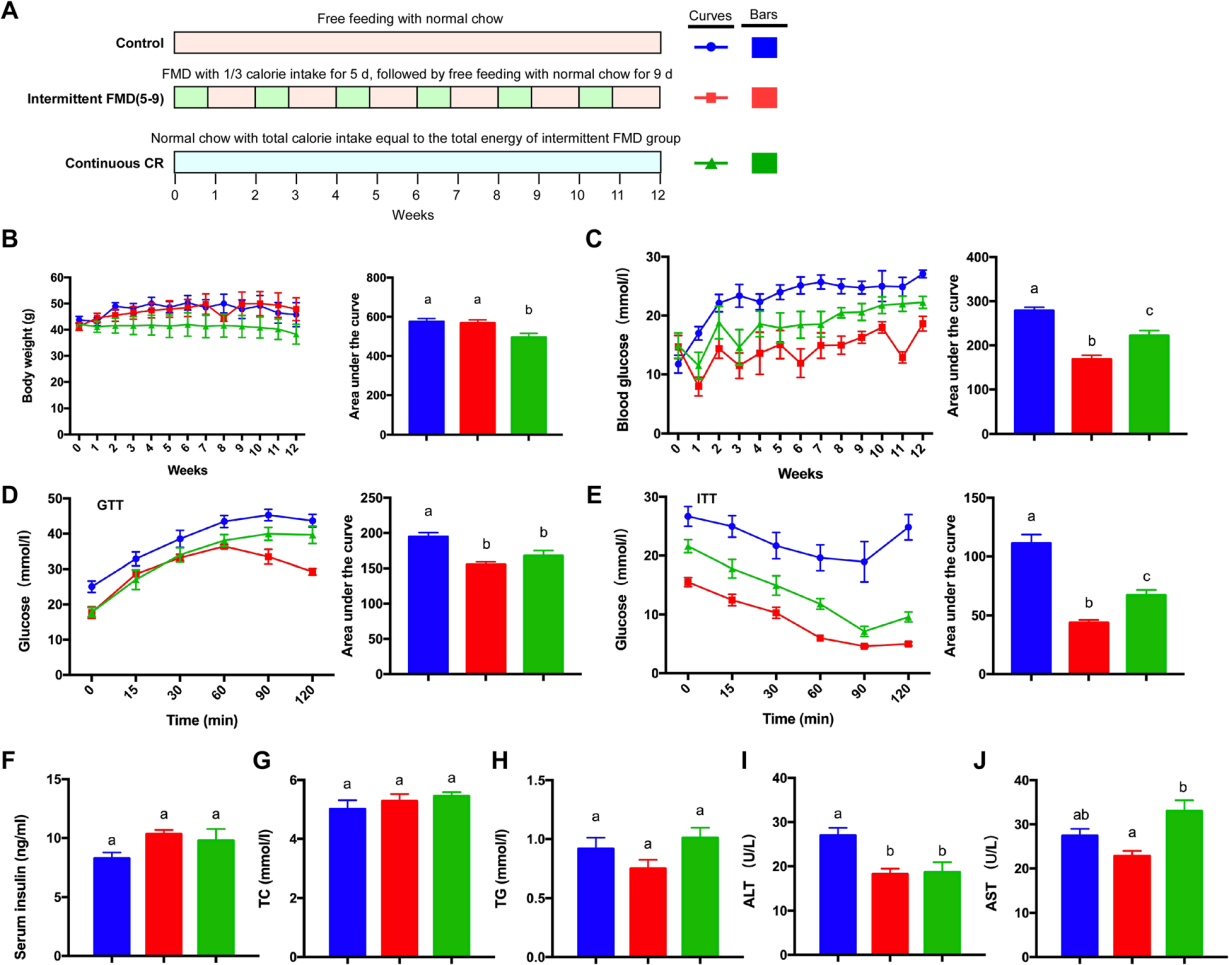
Comparison of 5–9 regime of intermittent fasting vs matched continuous CR in *db/db* mice. (**a**) Experimental design in *db/db* mice (n = 5 for each group). (**b**) Body weight. The area under the curve is shown on the right. (**c**) Blood glucose levels. Blood samples were collected on the last day of each cycle. Mice were fasted for 6 h (morning fasting) before glucose measurements. The area under the curve is shown on the right. (**d**, **e**) Glucose tolerance test and insulin tolerance test at week 13. The area under the curve is shown on the right. (**f**) Serum insulin levels under fed state. Blood samples were collected after mice were sacrificed. (**g**, **h**) Levels of total triglycerides and total cholesterol in the blood. (**i**, **j**) Blood ALT and AST levels of the mice. Data are expressed as the mean ± SEM, n = 5 per group. Significant differences were assessed by one-way ANOVA followed by the Student-Newman-Keuls test where appropriate

We next analyzed another regime of intermittent FMD together with the calorie-matched continuous CR. The *db/db* mice were divided into three groups (Fig. 2a). The control group had free access to normal chow. The intermittent FMD group was exposed to an alternating dietary pattern that consisted of FMD for 5 days and free access to normal chow for 9 days. The energy intake during FMD days was 1/3 of daily energy intake of the control group. The calorie-matched continuous CR group had normal chow with total energy intake equal to the intermittent FMD group (~ 76% of energy intake of the control group per day) (Fig. 2a). During the 12 weeks of intervention, the cumulative body weight of the continuous CR group was significantly lower than the weight of the other two groups (Fig. 2b), indicating continuous CR is better than intermittent CR in body weight reduction. The elevated fasting blood glucose level in *db/db* mice was significantly reduced by both intermittent FMD and continuous CR. However, intermittent FMD was better than continuous CR in reducing fasting blood glucose level (Fig. 2c). GTT revealed that both intermittent FMD and continuous CR significantly improved glucose tolerance (Fig. 2d). ITT showed that both intermittent FMD and continuous CR improved insulin sensitivity, while intermittent FMD performed better than continuous CR in this assay (Fig. 2e). The serum levels of insulin, TC, and TG were not significantly changed among the three groups of mice (Fig. 2f and h). However, the blood ALT level was reduced by both intermittent FMD and continuous CR (Fig. 2i), while the blood AST level was not significantly altered by either type of calorie restriction (Fig. 2j).

To rule out the possibility that observed difference in the study between intermittent FMD and continuous CR was caused by the FMD, we analyzed intermittent CR with normal chow. One group of intermittent CR with normal chow was added in the 2–5 regime experiment (Additional file 1: Figure S2A). The cumulative body weight was not altered by intermittent CR with normal chow (Additional file 1: Figure S2B). The decrease of fasting blood glucose level of intermittent CR with normal chow was the same as intermittent FMD, while better than continuous CR (Additional file 1: Figure S1C). In addition, the glucose tolerance and insulin sensitivity of the intermittent CR with normal chow group were not significantly different from the intermittent FMD group (Additional file 1: Figure S1D and E). Overall, these data indicated that intermittent FMD is identical to intermittent CR with normal chow to improve glycemic control in the *db/db* mice when using the 2–5 regime of fasting.

### Comparison of intermittent fasting and continuous CR on intervention of STZ-induced diabetic mice

**Fig. 3:**
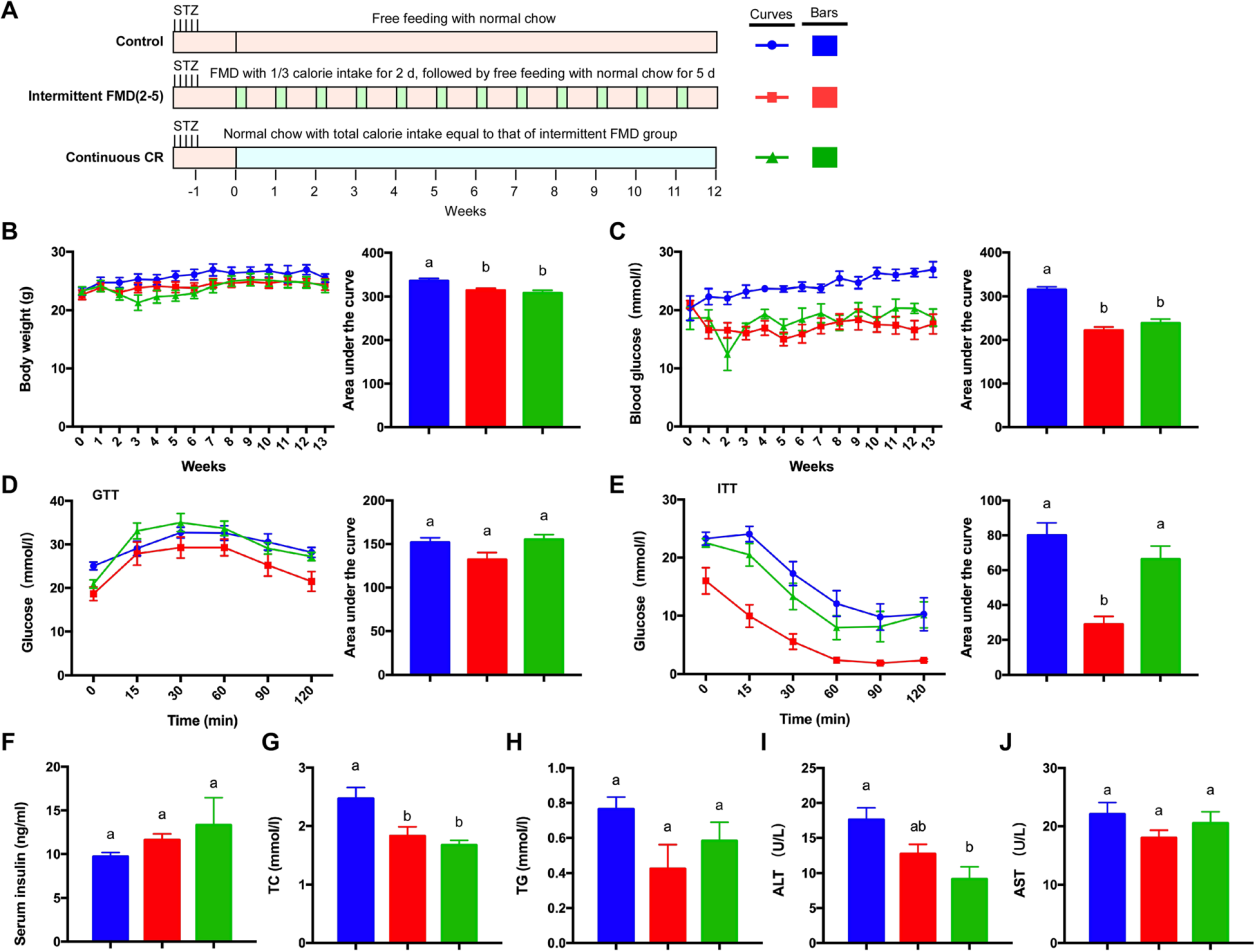
Comparison of 2–5 regime of intermittent fasting vs matched continuous CR in type 1 diabetic mice. (**a**) Experimental design with the STZ-treated mice (n = 5 for each group). STZ (40 mg/kg) was administered intraperitoneally for five consecutive days. An equal volume of citrate buffer was injected into control mice. (**b**) Body weight. The area under the curve is shown on the right. (**c**) Blood glucose levels. Blood samples were collected on the last day of each cycle. Mice were fasted for 6 h (morning fasting) before glucose measurements. The area under the curve is shown on the right. (**d**, **e**) Glucose tolerance test and insulin tolerance test at week 13. The area under the curve is shown on the right. (**f**) Serum insulin levels under fed state. Blood samples were collected after mice were sacrificed. (**g**, **h**) Levels of total triglycerides and total cholesterol in the blood. (**i**, **j**) Blood ALT and AST levels of the mice. Data are expressed as the mean ± SEM, n = 5 per group. Significant differences were assessed by one-way ANOVA followed by the Student-Newman-Keuls test where appropriate

To further explore the interventional effect of intermittent fasting or continuous CR on diabetes, we analyzed a type 1 diabetes (T1D) mouse model that was generated by STZ injection in C57BL/6 mice. We first analyzed the 2–5 regime of intermittent FMD together with the calorie-matched continuous CR (Fig. 3a). During 13 weeks of intervention, the cumulative body weight of the mice was significantly reduced by both intermittent FMD and continuous CR (Fig. 3b). The elevated fasting blood glucose level in the T1DM mice was significantly reduced by both intermittent FMD and continuous CR (Fig. 3c). GTT was not significantly altered by either type of fasting (Fig. 3d). However, ITT revealed that only intermittent FMD could significantly improve insulin sensitivity (Fig. 3e). However, the blood insulin level was not significantly different among the three groups of mice (Fig. 3f). Both intermittent FMD and continuous CR reduced the serum total cholesterol level (Fig. 3g), while the blood triglyceride level was not altered (Fig. 3h). The blood ALT level was significantly decreased by continuous CR (Fig. 3i), while the blood AST level was not changed (Fig. 3j).

**Fig. 4:**
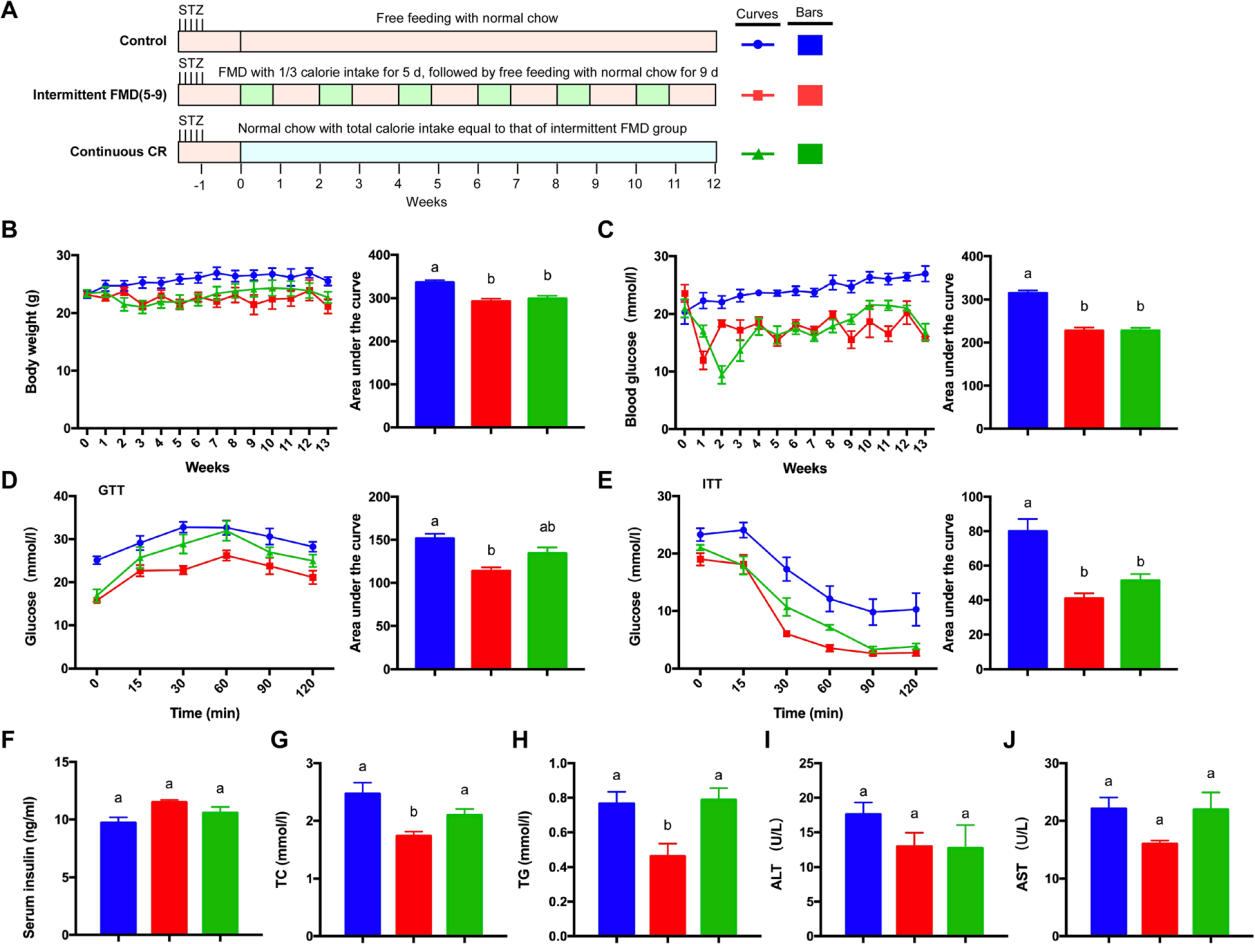
Comparison of 5–9 regime of intermittent fasting vs matched continuous CR in type 1 diabetic mice. (**a**) Experimental scheme with the STZ-treated mice (n = 5 for each group). STZ (40 mg/kg) was administered intraperitoneally for five consecutive days. An equal volume of citrate buffer was injected into control mice. (**b**) Body weight. The area under the curve is shown on the right. (**c**) Blood glucose levels. Blood samples were collected on the last day of each cycle. Mice were fasted for 6 h (morning fasting) before glucose measurements. The area under the curve is shown on the right. (**d**, **e**) Glucose tolerance test and insulin tolerance test at week 13. The area under the curve is shown on the right. (**f**) Serum insulin levels under fed state. Blood samples were collected after mice were sacrificed. (**g**, **h**) Levels of total triglycerides and total cholesterol in the blood. (**i**, **j**) Blood ALT and AST levels of the mice. Data are expressed as the mean ± SEM, n = 5 per group. Significant differences were assessed by one-way ANOVA followed by the Student-Newman-Keuls test where appropriate

We also investigated the 5–9 regime of intermittent FMD with the T1D mice together with the calorie-matched continuous CR (Fig. 4a). With this regime, the cumulatively body weight of the mice was significantly reduced by both intermittent FMD and continuous CR (Fig. 4b). The elevated fasting blood glucose level in the T1DM mice was significantly reduced by both intermittent FMD and continuous CR (Fig. 4c). GTT revealed that only intermittent FMD could significantly improve glucose tolerance (Fig. 4d). However, the insulin sensitivity of the mice was improved by both intermittent FMD and continuous CR (Fig. 4e). The blood insulin level was not altered in these mice (Fig. 4f). Only intermittent FMD reduced the total cholesterol and triglyceride levels of the mice (Fig. 4g and h). The blood levels of ALT and AST was not altered by either type of calorie restriction (Fig. 4i and j).

### Summary of the interventional efficacy of intermittent FMD vs continuous CR on both types of diabetes in the mice

**Fig. 5:**
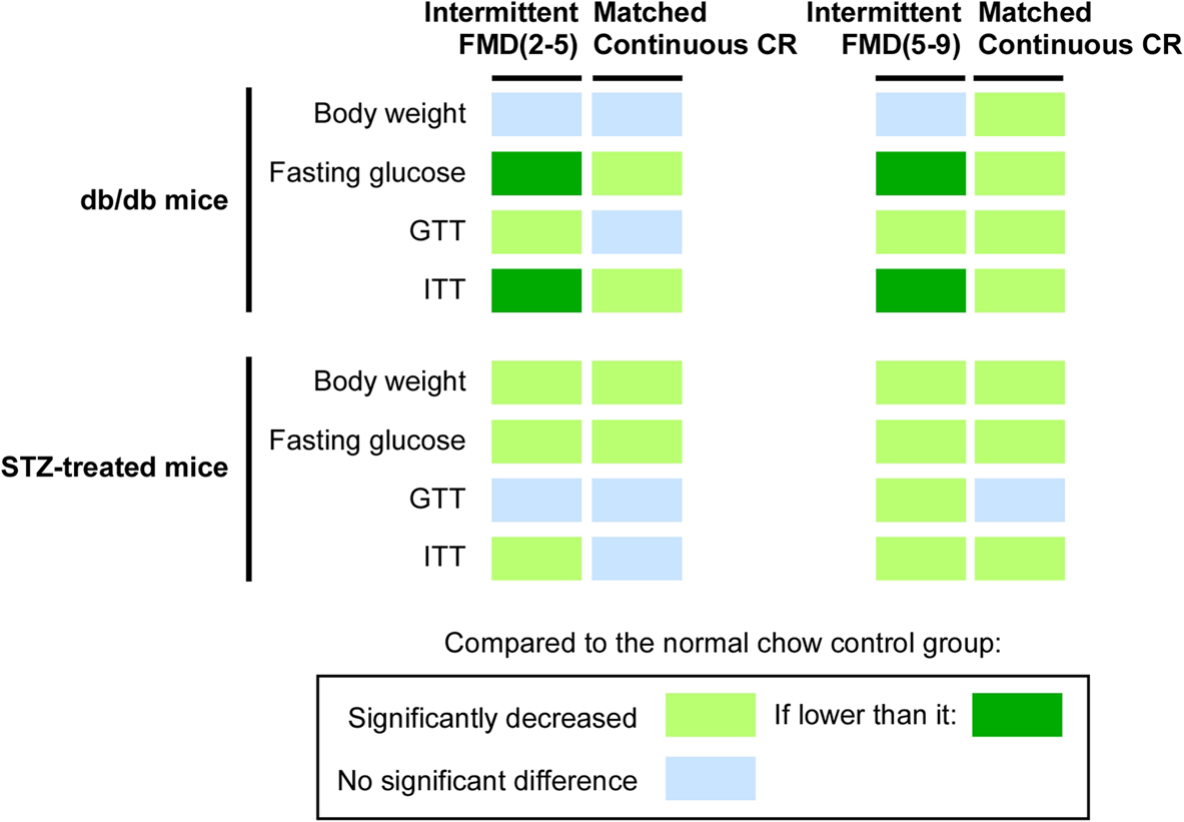
Summary of different regimes of fasting on glycemic control of different diabetic mouse models. Only the areas under the curves from Figs. 1, 2, 3 and 4 are used to compare the differences between different experimental groups

We summarized the animal data of our study (Fig. 5). In *db/db* mice, only continuous CR that matched the 5–9 regime of intermittent FMD significantly reduced the body weight. Both intermittent FMD and continuous CR significantly reduced the fasting blood glucose level. However, intermittent FMD performed better than continuous CR in this aspect. Both regimes of intermittent FMD significantly improved glucose tolerance, while only continuous CR matching the 5–9 regime of intermittent FMD improved it. In terms of insulin sensitivity, both intermittent FMD and continuous CR improved it, while intermittent FMD performed better than continuous CR. Overall, our data suggests that intermittent FMD has a better effect than continuous CR on reducing fasting blood glucose level and improving insulin sensitivity in *db/db* mice.

In the T1D mouse model, both intermittent FMD and continuous CR significantly reduced the body weight and the fasting blood glucose level (Fig. 5). The 5–9 regime of intermittent FMD improved glucose tolerance of the mice, while continuous CR had no effect in this aspect. The 2–5 regime of intermittent FMD but not its matched continuous CR improved insulin sensitivity. However, both 5–9 regime of intermittent FMD and the calorie-matched continuous CR improved insulin sensitivity. Overall, these results suggest that intermittent FMD and continuous CR are almost equally effective in improving the glycemic control in the T1D mice, except for that the 2–5 regime of intermittent CR performed better than continuous CR in improving insulin sensitivity and the 5–9 regime of intermittent FMD performed better than continuous CR in improving glucose tolerance.

We also directly compared the glycemic control data between the 2–5 and 5–9 protocols in both STZ-treated and *db/db* mice (shown in Additional file 1: Figure S6). For glycemic control, both intermittent FMD and continuous CR had the same effect to significantly lower the fasting blood glucose level in STZ-treated mice (Additional file 1: Figure S6A). However, in *db/db* mice, intermittent FMD protocols were better than their matched continuous CR groups (Additional file 1: Figure S6B). We next compared different parameters in *db/db* mice (Additional file 1: Fig. S7). Only continuous CR matching 5–9 regime gave rise to significant reduction of body weight (Additional file 1: Figure S7A). Consistent with the result of the fasting glucose level (Additional file 1: Figure S7B), both GTT and ITT were significantly better in intermittent FMD groups than their matched continuous CR groups (Additional file 1: Figure S7C and D). These comparisons, therefore, indicated that intermittent FMD performs better than continuous CR in improving glycemic control, glucose tolerance and insulin sensitivity in *db/db* mice.

### Immunofluorescent analysis of the pancreatic islets

**Fig. 6:**
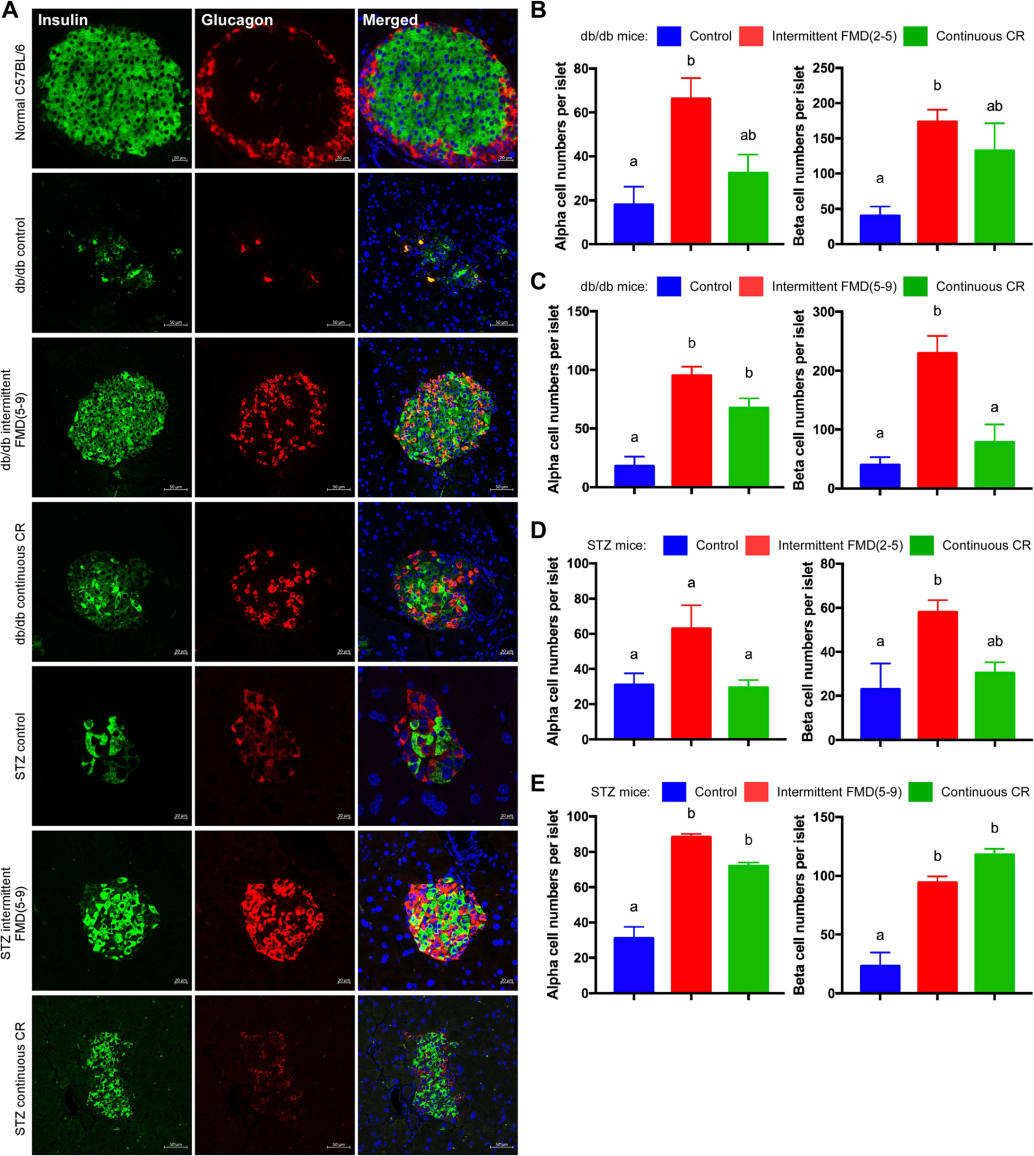
Immunofluorescence analysis of the pancreatic islets of the mice. (**a**) Representative immunofluorescence staining of pancreatic sections of normal C57BL/6 mice, *db/db* mice and STZ-treated mice with or without diet intervention (the whole data are shown in Additional file 1: Figures S3, S4 and S5). The pancreatic sections were stained with antibodies against insulin (green color) and glucagon (red color). The nucleus was stained with Hoechst 33342 (blue color). Scale bar, 50 μm. (**b**, **c**, **d**, **e**) Quantitation of β cells and α cells per islet based on the immunofluorescence staining data

Endocrine cells from pancreatic islets include α cells, β cells, δ cells, and pancreatic polypeptide (PP) cells, which produce glucagon, insulin, somatostatin, and pancreatic polypeptide respectively [14, 15]. The α cells commonly form a continuous mantle around the β cells in rodents [16]. We performed immunofluorescent staining with the pancreas sections of the mice to analyze both insulin-secreting β cells and glucagon-secreting α cells. In normal C57BL/6 mice, the majority of β cells were localized in the middle of the islets, surrounded by α cells in the peripheral zone (Fig. 6a and Additional file 1: Figure S3A). Most of the islets were markedly disrupted in both *db/db* and T1D mice with very few β and α cells present (Fig. 6b and Additional file 1: Figure S3B and C). Both regimes of intermittent FMD profoundly restored the islet structures. Most importantly, both 2–5 and 5–9 protocols of intermittent FMD could significantly increase the number of β cells in both *db/db* and STZ-treated mice (Fig. 6, Additional file 1: Figures S4 and S5). However, the distribution pattern of insulin-positive cells and glucagon-positive cells were not identical to the normal mice (Fig. 6, Additional file 1: Figures S4 and S5). In detail, some glucagon-positive cells were localized in the middle area in the islets instead of peripheral distribution in normal islets after diet intervention. Continuous CR could partially recover the insulin-positive cells in the islets, but not to the same degree as the intermittent FMD in the majority of the experiments (Fig. 6, Additional file 1: Figures S4 and S5).

## Discussion

This study analyzed the intervention activity of different protocols of intermittent calorie restriction. Our original protocol used a 7–7 regime in which the mice were given FMD containing 1/3 cal intake of the control mice for 7 days, followed by 7 days’ free eating [12]. In this study, we tried both 2–5 and 5–9 protocols in *db/db* and STZ-treated mice. The purpose of trying the intervention activity of different fasting protocols was to pave the way for future application in humans. We expect that shorter term of calorie restriction would be easier to be accepted by diabetic patients. Therefore, it is ideal to discover an effective intervention protocol that has the shortest period of intermittent fasting. It is noteworthy that the blood glucose level fluctuated in the first week in intermittent FMD groups (Figs. 1, 2, 3 and 4). This is likely due to the fact that these mice hadn’t been adapted to the new eating pattern.

One of the key findings of this study is that while both intermittent CR and continuous CR could significantly improve glycemic control, intermittent CR performed better than continuous CR in *db/db* mice (Figs. 1, 2 and 5). To our knowledge, this is the first study that directly compares the interventional activity of intermittent CR vs continuous CR within the same study in the mice. Intriguingly, it was found that intermittent fasting is better than continuous CR in improving longevity in *C. elegans* [17], consistent with our finding in certain degree. We postulate that intermittent CR accommodates better than continuous CR to the adaptation of most animals including humans during over millions of years of evolution. In nature, most animals commonly experience periodic long-term fasting interweaved with short-term feast. Accordingly, the metabolic machinery of most animals has adapted to such periodic dietary pattern. Only in recent years has this periodic dietary pattern in humans been replaced by an almost continuous eating pattern (such as three meals a day) due to the advances of agriculture and technology. Based on the viewpoint of evolution, it can be argued that periodic or intermittent CR should perform better than continuous CR to improve metabolic health and possibly aging in humans when over-nutrition has become a worldwide problem. As nicely summarized by Di Francesco and colleagues, periodic calorie restriction can mediate “periodic shifts of metabolic fuel sources, promotion of repair mechanisms, and the optimization of energy utilization for cellular and organismal health” [18]. With this idea in mind, research is urgently needed in human subjects to directly compare the activities of intermittent CR vs continuous CR in improving metabolic health and reducing risk factors of metabolic disorders.

Another interesting finding in our study is that loss of body weight is not necessarily associated with improvement of glycemic control in the mice. In our study, intermittent CR did not significantly reduce the body weight of *db/db* mice (Figs. 1, 2 and 5). However, continuous CR significantly decreased the body weight of the *db/db* mice when using 5–9 regime (Figs. 2 and 5). This discrepancy is likely caused by the fact that the mice using FMD might eat more when having free access to the food after stopping FMD (Additional file 1: Figure S1), while the food intake was constantly limited to the mice with continuous CR. However, improvement of glycemic control occurred in the mice with intermittent CR, without significant loss of body weight. Therefore, our observations indicate that intermittent CR has an independent effect to improve metabolic health in addition to the commonly believed beneficial effect of body weight reduction.

Our studies with the pancreatic islets provided additional evidence that intermittent CR might perform better than continuous CR in restoring the disrupted β cells in diabetic mice (Fig. 6). Based on the staining of insulin- and glucagon-positive cells in the islets, it appears that the 5–9 regime of intermittent CR performed the best in increasing the insulin-positive cells in the islets, better than the calorie-matched continuous CR (Fig. 6). Notably, there were many glucagon-positive cells in the central area of the islets in *db/db* mice and STZ-treated mice (Fig. 6 and Additional file 1: Figure S3), indicating a likely β-to-α transition in diabetic state. Interestingly, many cells in the islets were positive for both insulin and glucagon in *db/db* mice after intermittent FMD application (Fig. 6 and Additional file 1: Figure S4). This observation indicates there might be interconversion of α-to-β in the islets, likely explaining the interventional effect of intermittent CR in improving glycemic control in the diabetic mice. Nevertheless, our results indicated that intermittent CR could significantly restore the disrupted β cells in the diabetic mice.

Besides, other mechanisms might contribute to the interventional activity of intermittent CR. We analyzed the activity of insulin signaling in the skeletal muscle and liver as the ITT results (Figs. 1, 2, 3 and 4) indicated that insulin sensitivity was elevated by intermittent FMD. C57BL/6 mice were treated with STZ and then administrated with or with intermittent FMD for three weeks. The mice were fasted for 5 h in the morning and then injected with or without insulin. We analyzed the phosphorylation of AKT in both skeletal muscle and liver tissues. We found that intermittent FMD could significantly enhance insulin-stimulated AKT phosphorylation in these tissues (Additional file 1: Figure S8), indicating that insulin signaling is elevated by intermittent FMD.

In addition, our study revealed that intermittent CR was able to improve the glycemic control in STZ-treated mice, similar to previous reports [11, 12]. However, intermittent CR was almost as equally effective as continuous CR in the improvement of fasting glucose and insulin sensitivity (Figs. 3, 4 and 5). The discrepancy of intermittent CR in the improvement of glycemic control between *db/db* mice (in which it is better than continuous CR) and STZ-treated mice is likely caused by the difference in the pathogenesis of type 2 diabetes vs type 1 diabetes. In type 2 diabetes, both insulin resistance and β cells dysfunction contribute to the destruction of glucose homeostasis. However, the damage of β cells is the primary cause of type 1 diabetes. It will be of interest in the future to compare intermittent CR vs continuous CR using the NOD mouse model that mimics type 1 diabetes in humans better than STZ treatment. Nevertheless, our study has provided evidence that intermittent CR with FMD stands out as a promising strategy for the intervention of diabetes mellitus.

## Conclusions

Our study indicated that both intermittent CR and continuous CR can lower fasting blood glucose level in the diabetic mice, while intermittent CR is better than the latter in improving glucose homeostasis in *db/db* mice. Considering the effectiveness of intermittent CR in the intervention of diabetes in mice, this study would pave the way for carrying out similar studies in humans to control the progression of diabetes mellitus.

## Additional file

Additional file 1: **Figure S1.** Food intake of the mice. **Figure S2.** Comparison of 2–5 regime of intermittent fasting using normal chow with intermittent FMD and matched continuous CR in db/db mice. **Figure S3.** Fluorescent staining of pancreatic islets in control mice. **Figure S4.** Fluorescent staining of pancreatic islets in db/db mice after diet intervention. **Figure S5.** Fluorescent staining of pancreatic islets in STZ-treated mice after diet intervention. **Figure S6.** Comparison of glycemic control between intermittent FMD and continuous CR in db/db and STZ-treated mice. **Figure S7.** Comparison between intermittent FMD and continuous CR in db/db mice. **Figure S8.** Intermittent FMD enhances insulin signaling in the skeletal muscle and liver of the mice. (PDF 1400 kb)

## Data Availability

All data generated or analysed during this study are included in this published article and its Additional file.
