# Supplementary material for: Comparison of glycemic improvement between intermittent calorie restriction and continuous calorie restriction in diabetic mice
Source: Nutr Metab (Lond). 2019 Aug 28;16:60. doi: 10.1186/s12986-019-0388-x (PMC6714240; doi:10.1186/s12986-019-0388-x)
Supplement: Supplementary file 1 — Figure S1. Food intake of the mice. Figure S2. Comparison of 2–5 regime of intermittent fasting using normal chow with intermittent FMD and matched continuous CR in db/db mice. Figure S3. Fluorescent staining of pancreatic islets in control mice. Figure S4. Fluorescent staining of pancreatic islets in db/db mice after diet intervention. Figure S5. Fluorescent staining of pancreatic islets in STZ-treated mice after diet intervention. Figure S6. Comparison of glycemic control between intermittent FMD and continuous CR in db/db and STZ-treated mice. Figure S7. Comparison between intermittent FMD and continuous CR in db/db mice. Figure S8. Intermittent FMD enhances insulin signaling in the skeletal muscle and liver of the mice. (PDF 1400 kb) [file 12986_2019_388_MOESM1_ESM.pdf]

Supplemental Figures

Comparison of glycemic improvement between intermittent calorie restriction and continuous calorie restriction in diabetic mice

Siying Wei[1], Jingyu Zhao[1], Meijuan Bai[1], Chenchen Li[1,2], Lingling Zhang[1], and Yan Chen[1,2,*]

[1]CAS Key Laboratory of Nutrition, Metabolism and Food Safety, Shanghai Institute of Nutrition and Health, Shanghai Institutes for Biological Sciences, University of Chinese Academy of Sciences, Chinese Academy of Sciences, Shanghai, China, 200031
[2]School of Life Sciences and Technology, Shanghai Tech University, Shanghai, China, 200031

There are eight Supplemental Figures

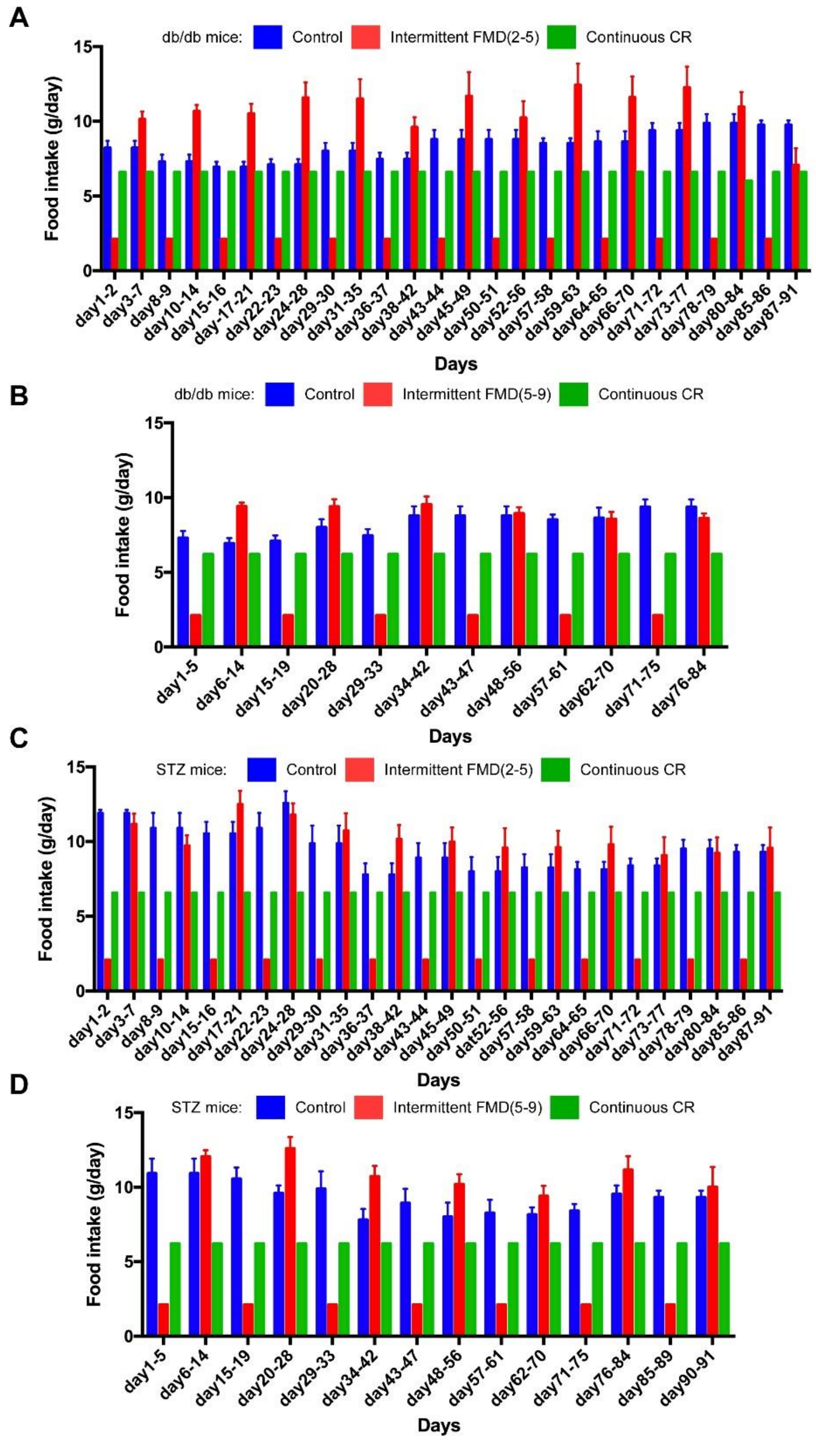

Figure S1. Food intake of the mice.

The food intake of the mice used in the experiments was recorded (n = 5 for each group). Note that the calorie intake of intermittent FMD group and continuous CR group were strictly controlled throughout the entire period of the experiment.

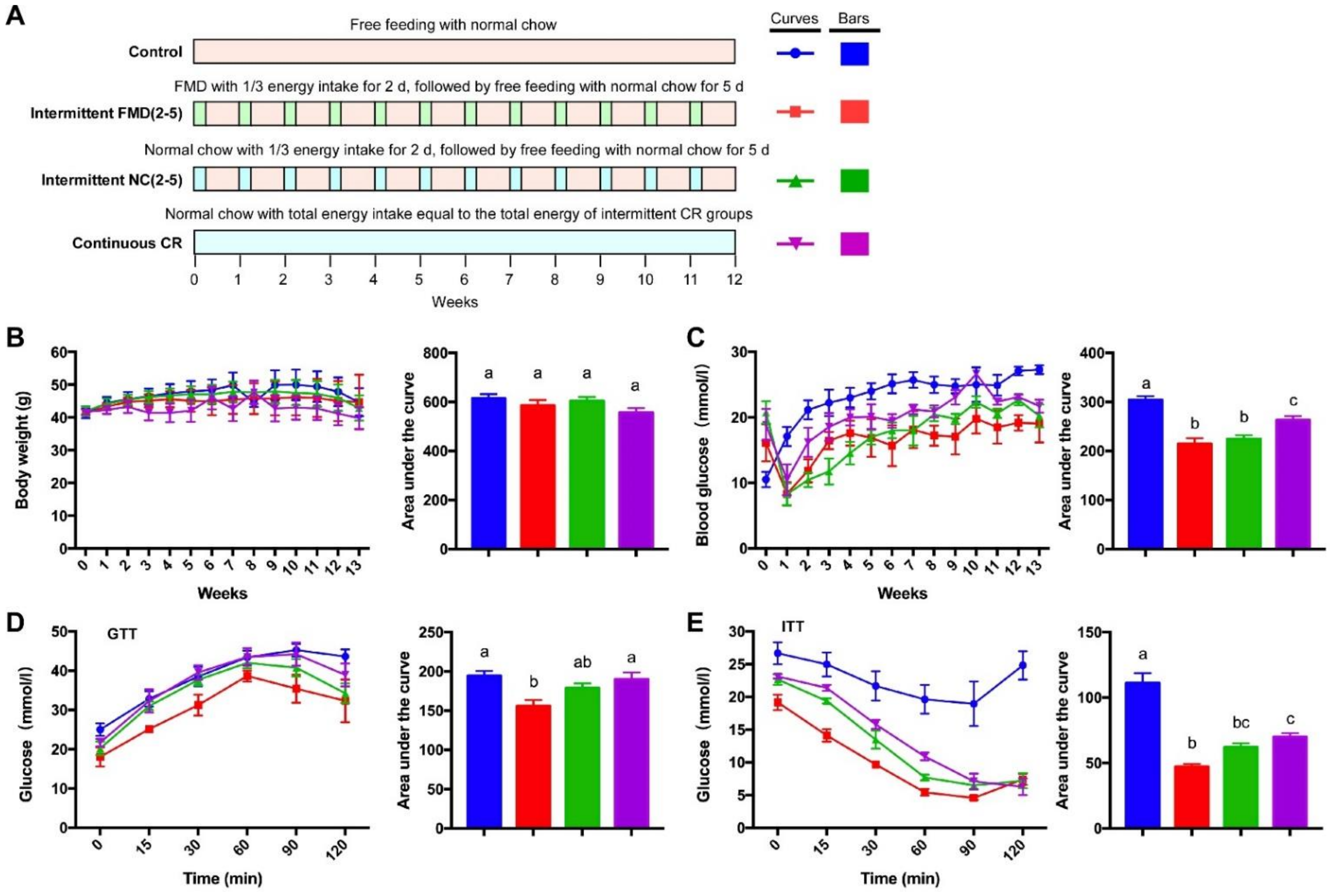

Figure S2. Comparison of 2-5 regime of intermittent fasting using normal chow with intermittent FMD and matched continuous CR in *db/db* mice

(A) Experimental design scheme with *db/db* mice (n = 5 for each group).

(B) Body weight. The area under the curve is shown on the right.

(C) Blood glucose levels. Blood samples were collected on the last day of each cycle. Mice were fasted for 6 h (morning fasting) before glucose measurements. The area under the curve is shown on the right.

(D, E) Glucose tolerance test (GTT) and insulin tolerance test (ITT) at week 13. The area under the curve is shown on the right.

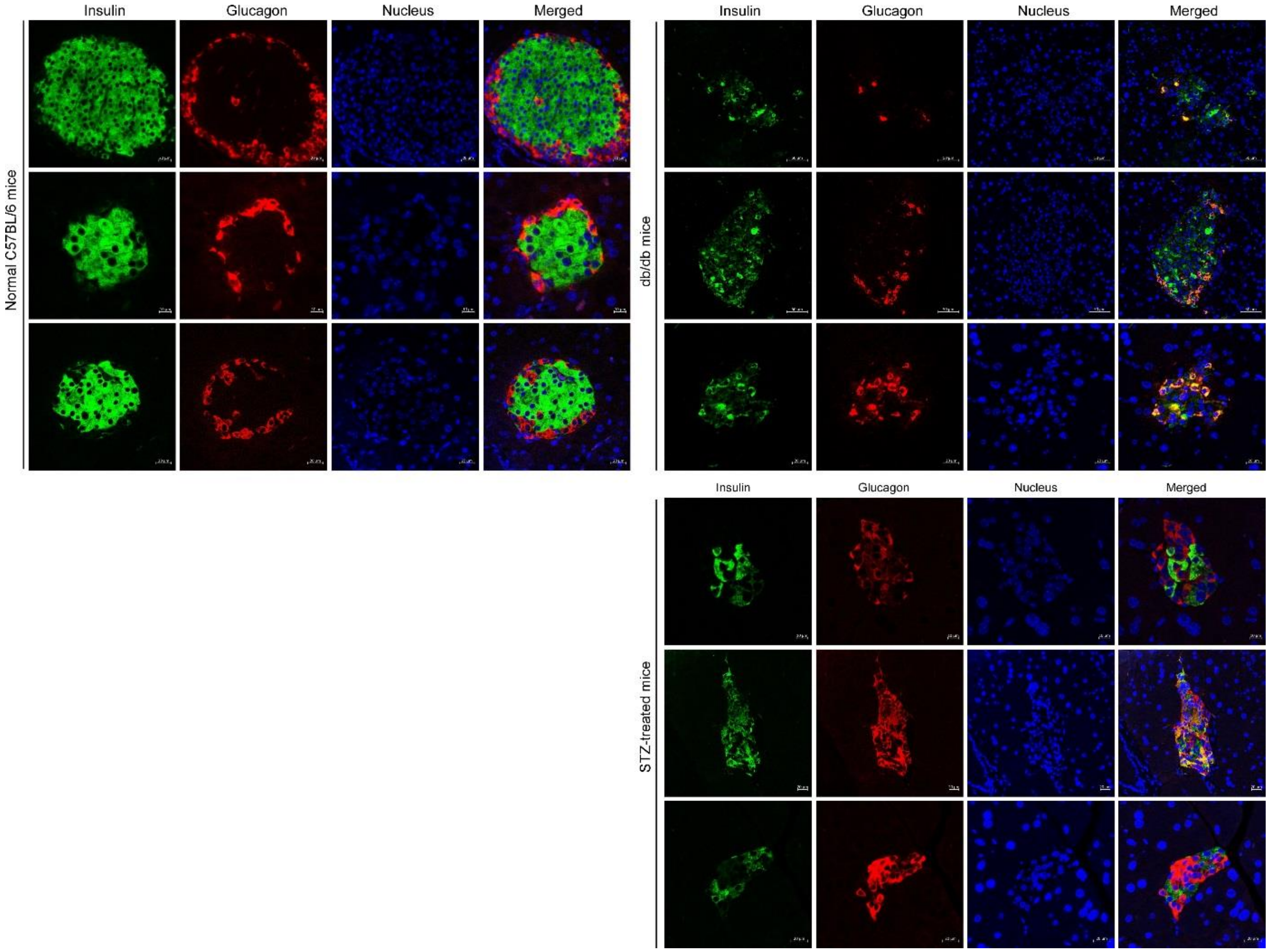

Figure S3. Fluorescent staining of pancreatic islets in control mice.

Pancreatic sections were used in immunofluorescent staining for insulin (green), glucagon (red) and nucleus (blue). The data from C57BL/6 mice, *db/db* mice and STZ-treated mice are shown here.

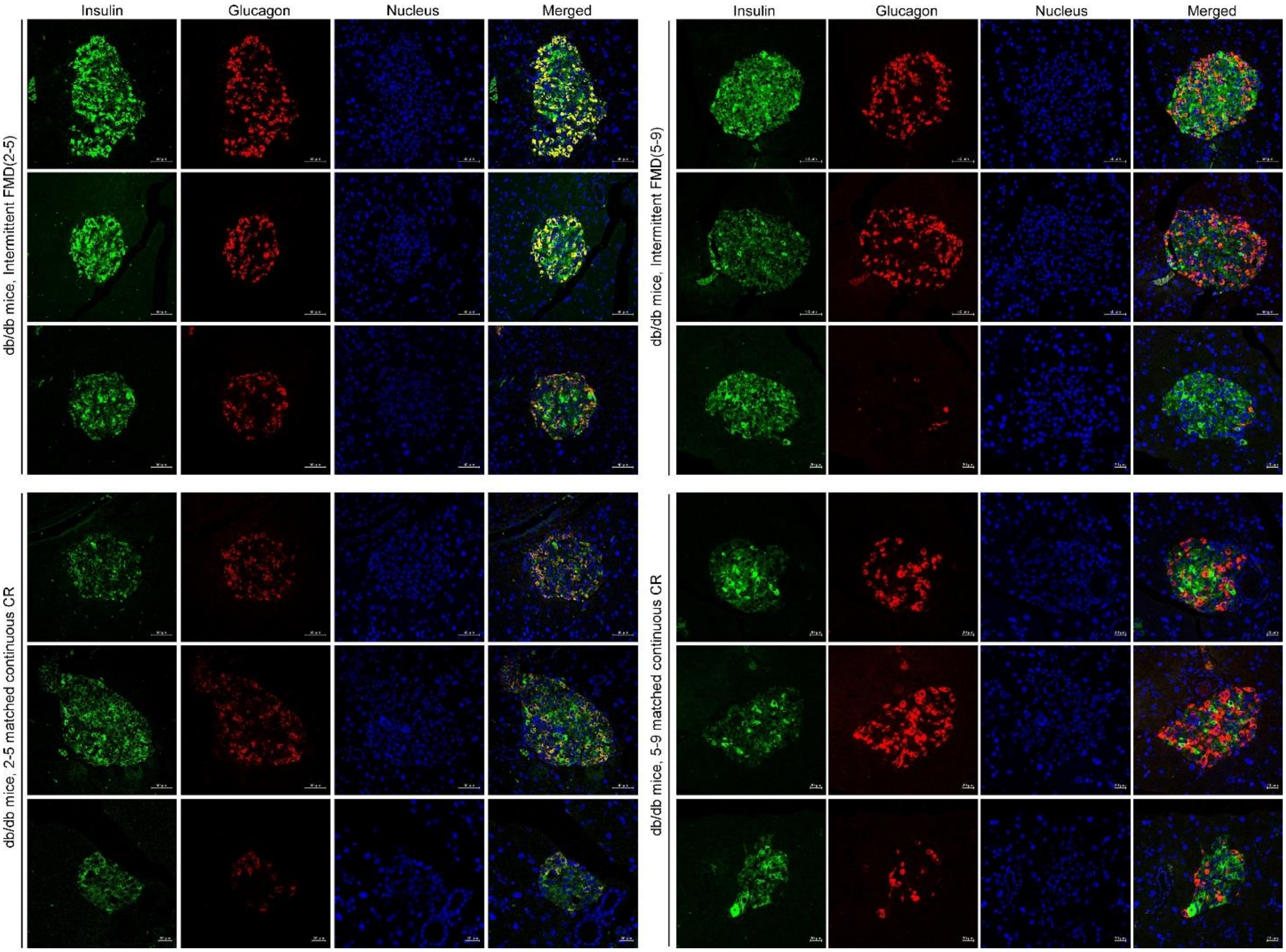

Figure S4. Fluorescent staining of pancreatic islets in *db/db* mice after diet intervention.

Pancreatic sections were used in immunofluorescent staining for insulin (green), glucagon (red) and nucleus (blue). The data from *db/db* mice administrated with different types of intervention are shown here.

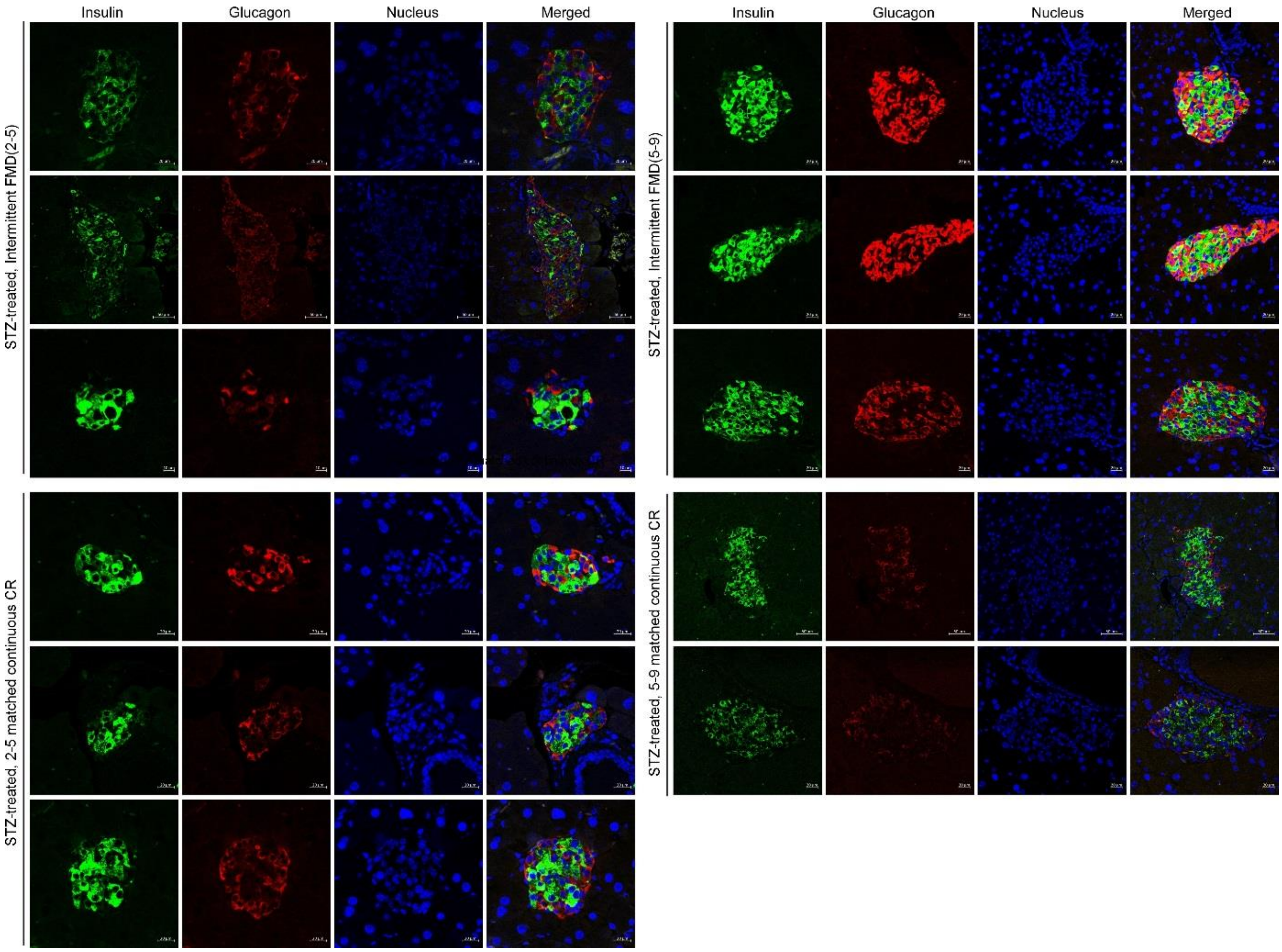

Figure S5. Fluorescent staining of pancreatic islets in STZ-treated mice after diet intervention.

Pancreatic sections were used in immunofluorescent staining for insulin (green), glucagon (red) and nucleus (blue). The data from STZ-treated mice administrated with different types of intervention are shown here.

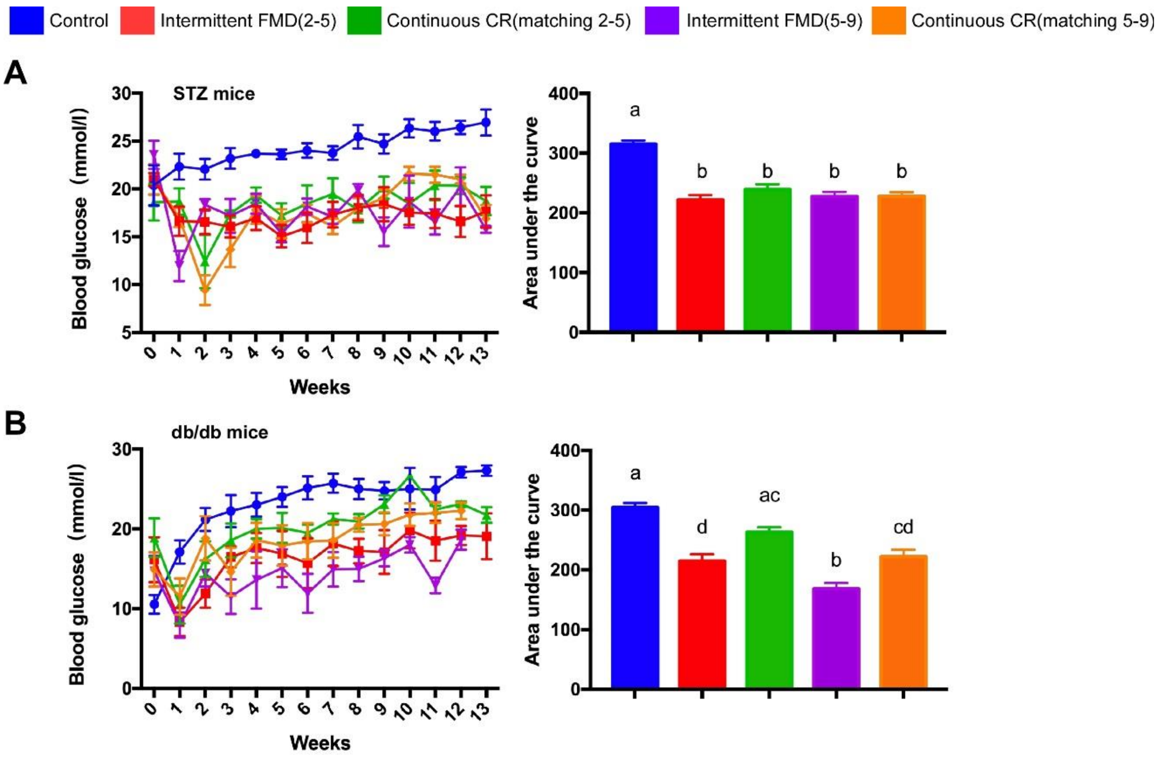

Figure S6. Comparison of glycemic control between intermittent FMD and continuous CR in *db/db* and STZ-treated mice.

Blood samples were collected on the last day of each experimental cycle. Mice were fasted for 6 h (morning fasting) before glucose measurement. The area under the curve is shown on the right.

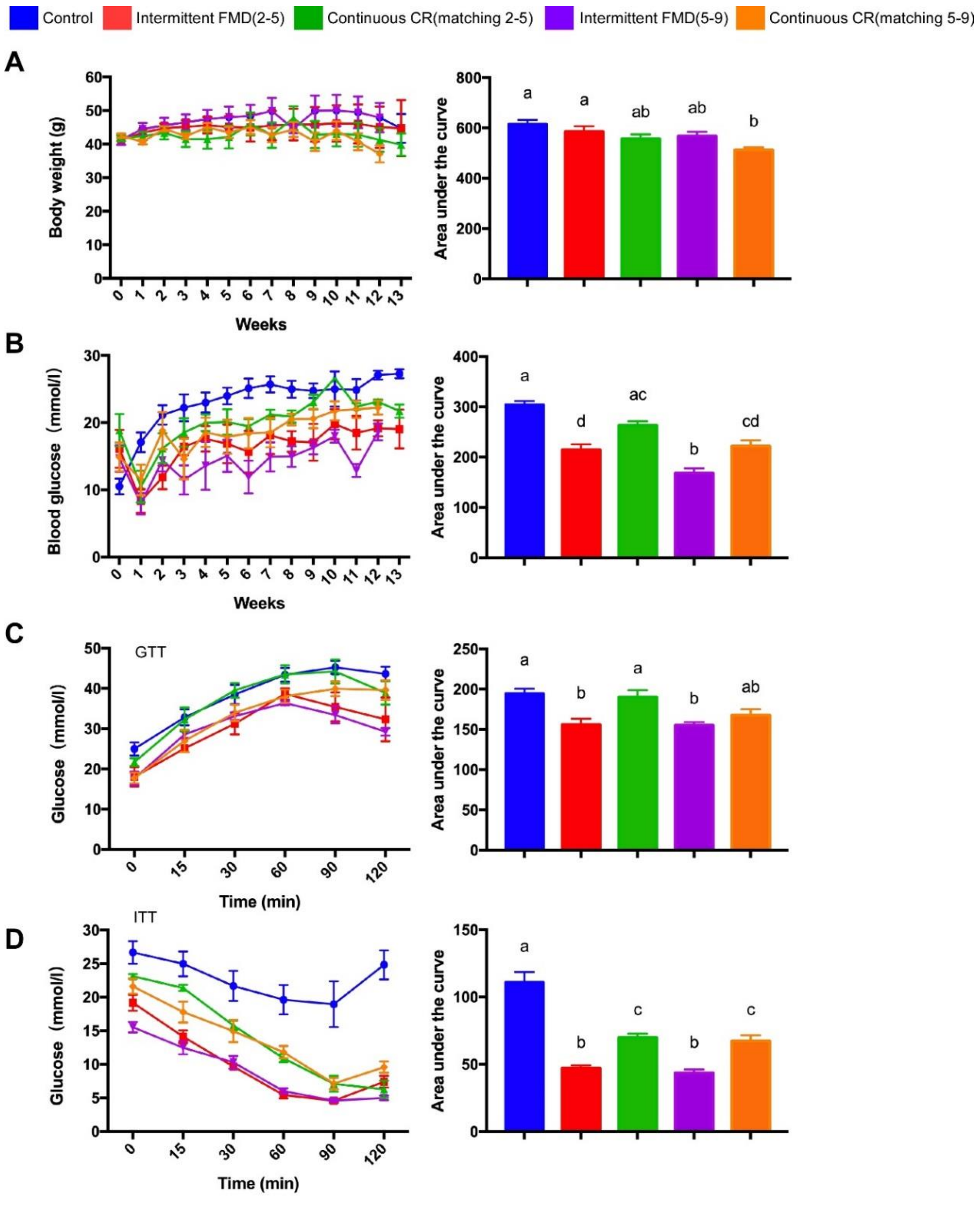

Figure S7. Comparison between intermittent FMD and continuous CR in *db/db* mice.
(A) Body weight. The area under the curve is shown on the right.
(B) Blood glucose levels. Blood samples were collected on the last day of each experimental cycle. Mice were fasted for 6 h (morning fasting) before glucose measurements. The area under the curve is shown on the right.
(C, D) Glucose tolerance test (GTT) and insulin tolerance test (ITT) at week 13. The area under the curve is shown on the right.

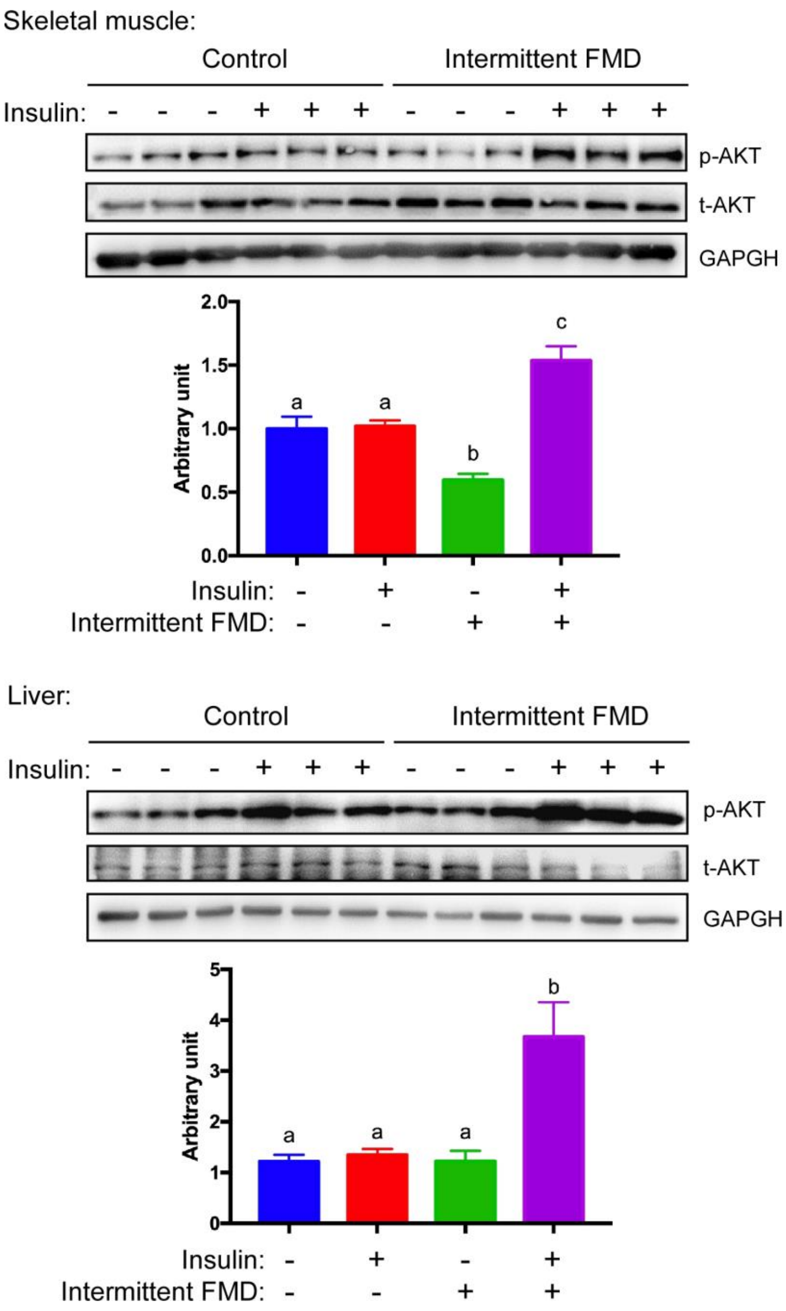

Figure S8. Intermittent FMD enhances insulin signaling in the skeletal muscle and liver of the mice

STZ-treated mice were administered with or without intermittent FMD for 3 weeks. The mice were fasted for 5 hours in the morning, followed by stimulation with PBS or insulin (4U per mice) for 8 minutes. The mouse tissues were used in Western blotting with the antibodies as indicated. p stands for phosphorylated protein and t for total protein. Quantification of the blot (shown as the ratio of p-AKT/t-AKT) is shown in the lower panel.
